# Protective Role of Sulodexide on Renal Injury Induced by Limb Ischemia-Reperfusion

**DOI:** 10.1155/2021/6629718

**Published:** 2021-01-30

**Authors:** Tao Yuan, Ni Yang, Wei Bi, Jinwen Zhang, Xueyan Li, Long Shi, Yang Liu, Xiang Gao

**Affiliations:** Department of Vascular Surgery, Second Hospital of Hebei Medical University, Shijiazhuang 050000, China

## Abstract

**Background:**

Though widely known as a potent antithrombin agent with protective effects on the kidney and other remote organs, it is currently ambiguous when it comes to sulodexide's function on ischemia-reperfusion (I/R) injury. With this research, we pursued to further explore how sulodexide exerts its influence on limb I/R injury, in which deleterious effects on the kidney were what we primarily focused on.

**Methods:**

We randomized twenty-four C57BL/6 male rats into three groups, namely, sham operation group (control group), I/R group, and sulodexide pretreatment group. Hematoxylin and eosin staining was applied for discovery of renal histological changes. Serum creatinine (Cr) and serum urea nitrogen (BUN) were measured. Apoptotic parameters were detected by the TdT-mediated dUTP Nick-End Labeling method. To what extent and levels that antiapoptotic and proapoptotic proteins were expressed could be sensitively revealed by immunohistochemistry assay. Lipid peroxidation product propylene glycol and inflammatory factors were examined by enzyme-linked immunosorbent assay. Additionally, an extracorporeal hypoxia-reoxygenation (H/R) model of human renal proximal tubule epithelial HK2 cells was established. Our targets lay in cell proliferation and apoptosis, and we used western blotting to reflect apoptosis-related gene expression.

**Results:**

The levels of serum BUN, Cr, and inflammatory factors in sulodexide-intervened rats manifested significant reduction when compared with the I/R group. Also, sulodexide could protect the kidney from histological changes and could effectively inhibit intraparenchymal apoptosis. Furthermore, adding 2 *μ*l/mL or 5 *μ*l/mL of sulodexide to H/R model cells in vitro gave rise to significant restoration of the degenerative proliferation capacity of the HK2 cells following H/R injury and late cellular apoptosis experienced dramatic reduction versus the H/R group. When treated with 5 *μ*l/mL of sulodexide at a dose of 10 mg/kg, the levels of the antiapoptotic proteins were increased, while the proapoptotic proteins showed opposite trends. Notable escalation on antiapoptotic protein expression level, in contrast with the opposite trends exhibited in proapoptotic proteins, was observed with 5 *μ*l/mL sulodexide pretreatment with the dosage being 10 mg/kg.

**Conclusion:**

Sulodexide can protect against kidney damage caused by I/R injury of the lower limbs by enhancing cell proliferation, inhibiting apoptosis, reducing inflammatory reactions, and scavenging oxygen free radicals.

## 1. Introduction

Ischemia-reperfusion (I/R) is a common phenomenon in clinical practice associated with high morbidity and mortality [1]. Due to its high incidence and devastating systemic effects [1], ischemia-reperfusion (I/R) injury has grabbed extensive attention in a host of clinical situations, as ischemia induces serious damage to not only the local organ but other involved organs as well, which is subsequently exacerbated by reintroduction of oxygen upon reperfusion [2]. In this process, a series of pathophysiological steps are strongly interacted with final postoperative death, among which distant multiple organ dysfunction is considered to be a fatal initiator [3–6].

In addition to remote injury in lung and intestinal tissues, acute kidney injury (AKI) also usually occurred following limb I/R injury [7–9]. Although efforts had been made to avoid or at least partly attenuate AKI, associated morbidity and mortality rates remain high over the decades [10]. Therefore, identifying novel preventive measures to lower the incidence of AKI and improve clinical outcomes is of urgent demand.

To date, even if awaiting to be fully clarified, the precise mechanisms underlying I/R injury have increasingly been disclosed, with inflammatory reaction, bursting release of reactive oxygen species and large scales of apoptotic cells generation predominantly composing this pathophysiological condition [11–13]. Moreover, the confirmed evidence that medications such as heparin [14, 15] may attenuate I/R injury exactly implies the crucial role played by microvascular thrombosis formation in this I/R injury-induced nonequilibrium status [12]. Therefore, clinically, it is imperative to identify pharmacological agents with multifunctional properties such as anticoagulation, anti-inflammatory, and antioxidant activities that may be effective treatments for AKI attributed to lower extremities ischemia-reperfusion injury.

Sulodexide, as a new antithrombotic agent, to be more precise, a glycosaminoglycan under very purification, has reportedly shown positive effects in many conditions that result from vascular surgery, with heparin sulfate and dermatan being its two major molecular components [16]. The pharmacological effects of sulodexide are made apparent by its capacity to repair the inner wall of injured blood vessels including by rebuilding the barrier structure of the vascular endothelial lumen surface and the glycocalyx and by restoring basic functions and selective permeability of the vascular endothelium and the ability to significantly reduce inflammatory factors, such as matrix metalloproteinase (MMP)-9, thereby lessening damage to the endothelium caused by inflammatory factors [17–21]. Also, with heparan sulfate and dermatan sulfate, respectively, possesses great affinity for antithrombin III and heparin cofactor II, the mutually strengthened anticoagulant effect makes sulodexide a dual antithrombotic agent [16, 22]. Moreover, blood rheology is improved by the drug's lipid-lowering effect triggered by activating lipoprotein esterase activity [16, 23]. Sulodexide may also exert positive effects in renal disease [24–27] by significantly reducing the level of proteinuria, preventing the onset of a nephropathy hypercoagulable state, inhibiting renal fibrosis, and protecting residual renal morphology and function. Our research aims to explore whether sulodexide could avoid or attenuate lower-limb I/R injury and kidney malfunction caused by this event and further investigate the associated pathophysiological mechanism.

## 2. Materials and Methods

### 2.1. Experimental Animals

Eighteen male C57BL/6 mice (8–10 weeks old, with weight ranging from 20 g–25 g) were obtained from the Experimental Animal Center of the Chinese Academy of Sciences (Beijing, China). All of the work was carried out in accordance with the regulations required by ethics committee of Hebei Medical University. The study animals were accommodated in a relatively homothermal and humidity-controlled room, where food and water was provided ad libitum.

### 2.2. Experimental Design

We randomly arranged the eighteen rats into three groups of six animals each (*n* = 6). All rats were intraperitoneally treated with 40 mg/kg of sodium pentobarbital (Sigma-Aldrich, St. Louis, MO, USA) and kept at 37°C. In the sham-operated control (sham) group, the femoral artery in the right leg was exposed and the incision was then closed without inducing I/R injury. In the group of I/R, the right femoral artery was also exposed and a rubber band was used to occlude the right hind femoral artery, thus inducing limb perfusion dificits for three hours, followed by band release to allow reperfusion for four hours. Finally, in the I/R + sulodexide group, exposure to rubber band application to limb ischemia for three hours was in combination with intravenous injection of 10 mg/kg of sulodexide; the block was then released for four hours to allow reperfusion. After seven hours, intraperitoneal administration of sodium pentobarbital injection at 100 mg/kg was implemented on the rat for euthanization.

### 2.3. Blood Collection and Detection

Prior to euthanasia, the right eyeball of each animal was obtained for 0.5 mL of the blood sample, allowed to stand for 15 minutes in a thermostatic water bath at 35°C, with rotating speed at 3,000 r/min for five minutes for centrifugation. The supernatant was then collected for following analysis: blood urea nitrogen (BUN) (Jiangcheng Biotechnology, Nanjing, China) and creatinine (Cr) (Jiangcheng Biotechnology) following the manuals.

The levels of MMP-9, IL-1, TNF-*α*, and IFN-*γ* in the serum were measured with enzyme-linked immunosorbent assay kits according to the manufacturer's instructions. Enzyme-linked immunosorbent assay was secondarily conducted following the manufacturer's guidance, to evaluate serum inflammatory parameters including levels of MMP-9, interleukin (IL)-1, tumor necrosis factor (TNF)-*α*, and interferon (IFN)-*γ*.

### 2.4. Determination of Renal Oxidative Stress Biomarkers

Renal homogenates were prepared according to the method of Wang et al. [28].

### 2.5. Renal Histology

Immediately after organ procurement, the color, elasticity, degree of swelling, exudation, and the degrees of necrosis and adhesion were assessed and photographed. 4% paraformaldehyde was used to immerse and fix the renal tissue, which was then embedded in paraffin. Having been exposed to the above process, tissue sections measuring 5 *μ*m were stained with hematoxylin and eosin and examined for the renal morphology changes with a light microscope. Findings of interest included the degree of tissue edema, the destruction of nephron structural integrity, the accumulation of inflammatory cells, and the shedding of red blood cells.

### 2.6. Culture and Maintenance of HK2 Cells

HK2 cells, deriving from human renal proximal tubule epithelial cell line, were cultured in an extracellular matrix (ECM) medium (Gibco Laboratories, Gaithersburg, MD, USA) with fetal bovine serum at concentration of 10% contained (Gibco Laboratories).

### 2.7. Establishment of the In Vitro Hypoxia/Reoxygenation (H/R) Model of HK2 Cells

We model the cells to be victim of H/R injury when the growth was close to the confluent state; the number of cells was about 1 × 10^6^. The culture medium was replaced by a sugar-deficient solution (containing 1.8 mmol/L of CaCl_2_, 0.9 mmol/L of NaH_2_PO_4_, 6.0 mmol/L of NaHCO_3_, 20 mmol/L of HEPES, 1.2 mmol/L of MgSO_4_, 98.5 mmol/L of NaCl, and 10 mmol/L of KCl; pH: 6.8). Smooth gaseous influx of 5% CO_2_ combined with 95% N_2_ conteneously flowed into the sterilized oxygen-demanding box, where the culture dish was located, until there was less than 0.1% of oxygen inside the box and such environment should be maintained for one hour. Then, the culture medium was replaced by a simulated reperfusion solution (containing 5.0 mmol/L of KCl, 20 mmol/L of NaHCO_3_, 129.5 mmol/L of NaCl, 55 mmol/L of glucose, 0.9 mmol/L of NaH_2_PO_4_, 1.2 mmol/L of MgSO_4_, 1.8 mmol/L of CaCl_2_, and 20 mmol/L of HEPES; pH: 7.4), followed by incubation with 95% O_2_ and 5% CO_2_ for reoxygenation for 24 hours.

### 2.8. Cell Treatment

The experimental cells were divided into the following four groups: control, H/R, 2 *μ*l/mL of sulodexide + H/R and 5 *μ*l/mL of sulodexide + H/R. Being treated with 2 *μ*l/mL or 5 *μ*l/mL of sulodexide before reoxygenation was done where cells in sulodexide treatment groups distinguish from others. Repeatability was ensured with as much as three times of implementation on all experiments.

### 2.9. Sulforhodamine Experiments

SRB experiments were performed as previously described [29].

### 2.10. MTT Experiments to Detect Cell Viability

Twenty thousand treated HK2 cells were plated into a 96-well plate (*n* = 6) each of which was added with 20 mL of MTT (5 mg·mL). Later another four hours was spent for incubation of the plates at 37°C. The next step was to remove the MTT and to dissolve formazan crystals for 10 minutes in 150 mL of DMSO at 37°C. Measurement of absorbance was realized by a microplate reader at 570 nm.

### 2.11. Apoptosis Detection

Apoptosis (annexin V and PI double staining) was detected by flow cytometry according to the manufacturer's protocols (BD Pharmingen™, BD Biosciences, San Diego, CA, USA). Following the guidance of rules drawn by the manufacturer, staining cells with annexin V-FITC and PI was used to quantitatively evaluate apoptotic cells, while flow cytometry for detection (BD Pharmingen™; BD Biosciences, San Diego, CA, USA).

### 2.12. Reverse Transcription and Quantitative Polymerase Chain Reaction (PCR) to Detect Messenger RNA (mRNA) Expression

The whole spectrum of RNA was extracted using TRIzol reagent (Thermo Fisher Scientific) and reversely transcribed as previously referred. The SYBR green (Takara, Dalian, China) method and IQ5 real-time PCR detection system (BioRad Laboratories, Hercules, CA, USA) were used for real-time PCR. The primer sequences were as follows: BAX, forward: CCTTTTCTACTTTGCCAGCAAAC; BAX, reverse: GAGGCCGTCCCAACCAC; BCL-2, forward: TCCGCATCAGGAAGGCTAGA; BCL-2, reverse: AGGACCAGGCCTCCAAGCT; caspase-3, forward: AGAACTGGACTGTGGCATTGAG; caspase-3, reverse: GCTTGTCGGCATACTGTTTCAG; BCL-xL, forward: CATGGCAGCAGTAAAGCAAG; BCL-xL, reverse: TAGAGTTCCACAAAAGTATC; BAD, forward: AAGGCTTGGTCCCATCGGAAGTTT; BAD, reverse: TTAACATTTGGTAGTGAGCACGGCCC; GAPDH, forward: TGCACCACCAACTGCTTAGC; and GAPDH, reverse: GGCATGGACTGTGGTCATGAG.

### 2.13. Western Blot

As has been introduced, western blotting was orderly performed [28]. The antibodies, sourced from either Abcam (Cambridge, England) or Cell Signaling Technology (CST) (Danvers, MA, USA), were as follows: BAX, Abcam cat no. ab182734; BCL-2, Abcam cat no. ab692; caspase-3, CST cat no. 9662; BCL-xL, CST cat no. 2764; BAD, Abcam cat no. ab32445; *β*-actin, Abcam cat no. ab8227; goat antimouse horseradish peroxidase secondary antibody, Abcam cat no. ab47827; goat antirabbit horseradish peroxidase secondary antibody, Abcam cat no. ab7090; and enhanced chemiluminescence (ECL) substrate luminescent liquid, Abcam cat no. 133406.

### 2.14. Statistical Analyses

All of the data are presented as mean ± standard error of the mean. The statistical significance of parameters was assessed by one-way analysis of variance combined with Tukey's post hoc test using the Graph Pad Prism software version 5 (Graph Pad Inc., La Jolla, CA, USA). A *p* value of less than 0.05 was considered to be statistically significant.

## 3. Results

### 3.1. In an In Vivo Rat Model, Sulodexide Treatment Ameliorated Renal Dysfunction Caused by Hind-Limb I/R Injury

**Figure 1:**
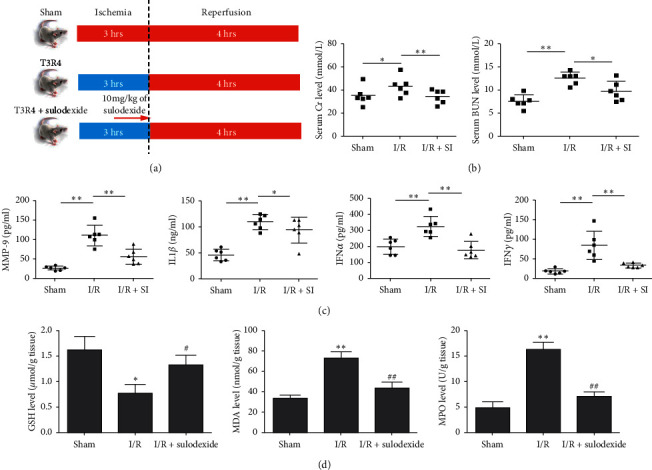
In an in vivo rat model, sulodexide treatment ameliorates renal dysfunction caused by hind-limb I/R injury. (a) A schematic to show hind-limb I/R injury and sulodexide intervention. (b) Evaluation of Cr and BUN levels in serum samples. (c) Evaluation of MMP-9, IL-1*β*, TNF-*α*, and IFN-*γ* levels in the animals' serum samples. (d) Assessment of glutathione, MDA, and MPO expression levels in renal parenchyma. The data were statistically analyzed by Student's *t* test. ^*∗*^, *P* < 0.1, ^*∗∗*^, *P* < 0.01, and ^*∗∗∗*^, *P* < 0.001.

Classic biomarkers were detected to assess the correlation between the renoprotection conferred by sulodexide under the circumstance of I/R injury and inflammation and oxidative stress. Firstly, an in vivo hind-limb I/R injury model was established. As shown in Figure 1(a), the mice were randomly allocated into three groups and each group was treated as described in the Materials and Methods section. As expected, the Cr and BUN levels in serum were significantly increased following hind-limb I/R treatment but attenuated by sulodexide treatment (Figure 1(b)). As Figure 1(a) illustrates, preliminarily, the randomly arranged three groups of animals were treated as afore mentioned in the Materials and Methods chapter, in which way we progressively established an in vivo hind-limb I/R injury model. Aligning with our previous expectation, attenuation of marked increase attributed to hind-limb injury was incurred by sulodexide treatment (Figure 1(b)), while similar trends were manifested in Figure 1(c), where dramatic elevation to various degrees on inflammatory factors such as MMP-9, IL-1*β*, TNF-*α*, and IFN-*γ* in serum was closely followed by significant dropping down by sulodexide. The MDA level and myeloperoxidase (MPO) activity in renal tissue also exhibited the same results as the above factors, while the glutathione level in renal tissue was significantly declined following hind-limb I/R treatment but would be improved by sulodexide treatment (Figure 1(d)). In summary, these results suggested that such dysfunction could be ameliorated using sulodexide partially by anti-inflammation and antioxidant stress.

### 3.2. Renal Histological Changes Occurred Following Hind-Limb I/R Injury in In Vivo Rat Models

**Figure 2:**
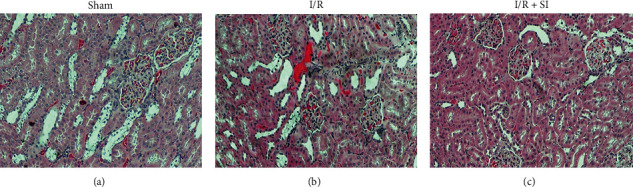
Hematoxylin and eosin staining of renal tissue from the rat with indicated treatment. (a) Sham-group rats. (b) Rats with hind-limb I/R injury. (c) Rats received I/R injury + sulodexide (10 mg/kg). Tissue sections were stained with hematoxylin and eosin. Scale bar, 100 *μ*m.

Hematoxylin and eosin staining of renal tissue from the I/R group in comparison with that from sham rats revealed that the renal tissue of the former group had suffered varying degrees of damage, including tissue edema, destruction of the nephron structural integrity, aggregation of inflammatory cells, and shedding of red blood cells. Yet sulodexide's promising potential to significantly ameliorate this damage was evidently bolstered by the contrary tendency after its application (Figure 2).

### 3.3. Renal-Cell Apoptosis Was Alleviated in I/R Injury Rats with Sulodexide Pretreatment

**Figure 3:**
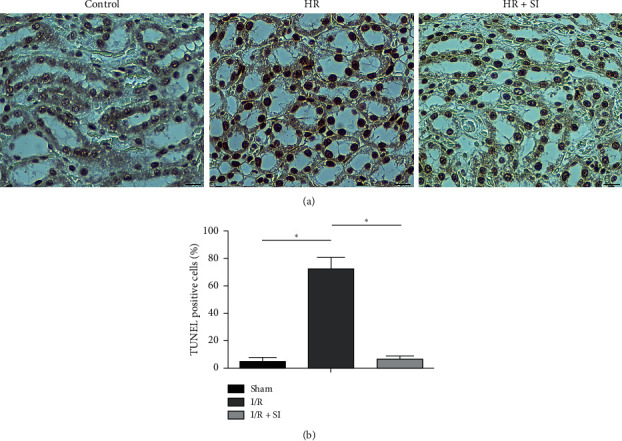
Renal tissues were assessed regarding apoptosis with the TUNEL method. (a) The proportion of cells towards apoptosis was increased significantly following hind-limb I/R injury, yet sulodexide intervention could effectively inhibit the occurrence of apoptotic cells. Scale bar, 50 *μ*m. (b) Statistical result. All of the data were statistically analyzed by Student's *t* test. ^*∗*^, *P* < 0.05.

**Figure 4:**
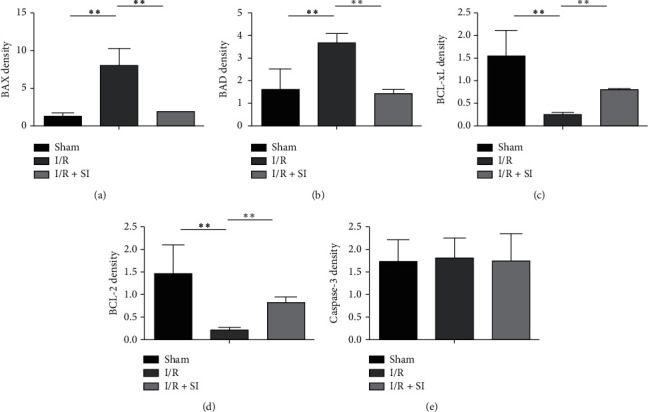
The expression of apoptosis and antiapoptosis proteins of renal tissues in in vivo rat models with western blotting. Hind-limb I/R injury in rats contributed to substantial downregulation in BCL-2 and BCL-xL expression, and correspondingly an uptrend in the expression levels of BAX and BAD. (a) BAX, (b) BAD, (c) BCL-xL, (d) BCL-2, and (e) caspase-3. All of the data were statistically analyzed by Student's *t* test. ^*∗∗*^, *P* < 0.01 and ^*∗∗∗*^, *P* < 0.001.

TdT-mediated dUTP Nick-End Labeling (TUNEL) assays on the kidney sections was conducted for deeper exploration on whether antiapoptosis process was involved in sulodexide's protective role under I/R injury. Administration of sulodexide remarkably reversed I/R injury induced growth in cell apoptosis compared with rats in the control group (Figure 3). Furthermore, to what extent the proapoptosis proteins BAX and BAD and the antiapoptosis proteins BCL-2 and BCL-xL expressed, together with the activity of caspase-3, were simultaneously tested as well. Here, it was observed that I/R injury led to a substantial decrease in the expression levels of BCL-2 and BCL-xL and an increase in the expression levels of BAX and BAD as indicated in Figure 4, although the total caspase-3 was not changed (Figure 4). Corresponding with precedent findings, BCL-2 and BCL-xL underwent explicit retardation on their expression, while opposite manifestation was seen in BAX and BAD, with the level of caspase-3 remaining stable (Figure 4). Taken together, these results suggest that renal-cell apoptosis in hind-limb I/R injury rats could be effectively ameliorated by sulodexide intervention.

### 3.4. Sulodexide Attenuated H/R-Induced Cellular Growth Arrest in HK2 Cells

**Figure 5:**
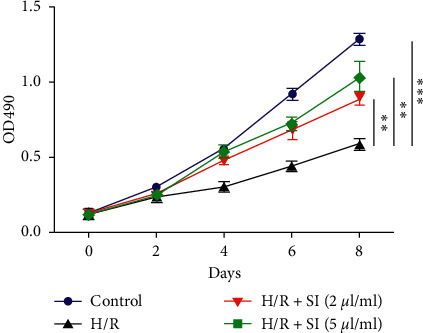
Sulodexide administration blocks H/R-induced HK2 cell growth arrest, which was determined by MTT assay. All data are shown as mean ± standard error of the mean. All of the data were statistically analyzed by Student's *t* test. ^*∗∗*^, *P* < 0.01 and ^*∗∗∗*^, *P* < 0.001.

In In Vitro H/R models on HK2 cells, we established that following the previous description, cell survival was subsequently detected to confirm the positive effects that sulodexide exerted on cellular growth arrest. Unsurprisingly, the suppression of cell viability induced by H/R treatment was blocked by sulodexide, whose noteworthy protective capacity against H/R injury was strongly indicated (Figure 5).

### 3.5. Sulodexide Attenuated H/R-Induced Cellular Apoptosis in HK2 Cells

**Figure 6:**
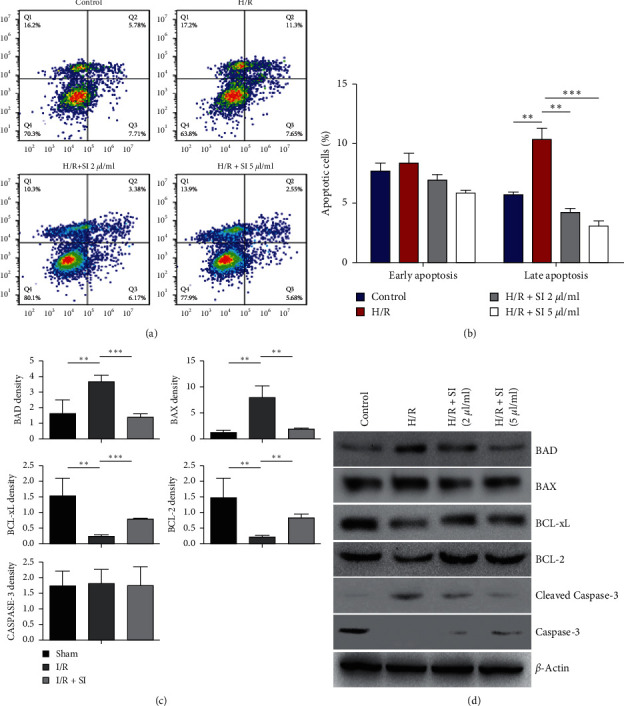
Sulodexide protects HK2 cells against H/R-induced apoptosis. (a) Representative image to show the early and late apoptotic cells in the aforementioned groups. (b) Quantification of apoptotic cells in HK2 cells with H/R and sulodexide treatment in early and late stages. (c) Quantitative PCR to directly reflect the expression levels of BAD, BAX, BCL-xL, BCL-2, and caspase-3. (d) The protein levels of BAD, BAX, BCL-xL, BCL-2, and caspase-3 visualizedly revealed by western blotting. ^*∗∗*^, *P* < 0.01 and ^*∗∗∗*^, *P* < 0.001.

To further determine the effects of sulodexide on H/R-induced cell apoptosis, we completed annexin V and PI double staining following with flow cytometry to determine HK2 cell apoptosis rates with indicated treatment. The effects of sulodexide on H/R-triggered HK2 cell apoptosis and necrosis was more accurately illuminated by annexin V/PI double staining, following flow cytometry to examine the apoptosis ratio. As shown in Figures 6(a) and 6(b), the significant late apoptosis (annexin V+/PI+) was pronouncedly inhibited by sulodexide, whereas sulodexide had little effect on early apoptosis (annexin V+/PI−). H/R treatment induced diving on BCL-2 and BCL-xL expression levels emerged with employment of quantitative real-time PCR (qPCR) and western blot analysis, but sulodexide made it possible to be preserved. Meanwhile, at the same time, proapoptotic proteins BAX, BAD, and caspase-3 exhibited the opposite trend (Figures 6(c) and 6(d)).

## 4. Discussion

In addition to the damage that occurs in local tissues receiving hind-limb I/R injury, distant organs can also be affected. The kidney, as one example, can experience AKI [4, 5]. Thus far, there is no clinically precise and significant treatment. Moreover, the exact underlying molecular mechanism still remains unknown. In combination with previous relevant literature reports, inflammation, oxidative stress, apoptosis, and the generation of microvascular thrombosis play pivotal roles in the pathogenesis of I/R injury [11–13]. Given that sulodexide is a drug with multifunctional properties covering anticoagulation, anti-inflammation, and antioxidant stress, in current study, the therapeutic effects of sulodexide on renal injury following hind-limb I/R injury and the latent mechanisms were determined.

Characterized with suppressed oxidative reaction, blocked cascade amplifying inflammation, preserved cell viability, and reversed histological and functional renal changes, sulodexide exerted highly protective capacity against hind-limb I/R injury in our research. Moreover, the direct attenuation towards cell growth arrest and apoptosis is H/R-induced HK2 cells also exactly matched with our prediction. Based on this evidence, we believe that the renoprotective effects of sulodexide on I/R injury of the lower limbs may be mediated via the above mechanisms, with sulodexide presented as a promising therapeutic drug.

Apart from its anticoagulation property, sulodexide also possesses anti-inflammatory [17–21] and antioxidative [30] effects. Supplementarily, the present study demonstrated that, in vitro, levels of inflammatory factors such as MMP-9, IL-1*β*, TNF-*α*, and IFN-*γ* levels in serum all increased to varying degrees following I/R treatment and were attenuated by sulodexide treatment. Meanwhile, the MDA level and MPO activity in renal tissues showed similar trends as those of the above factors, while the glutathione level in renal tissues significantly declined following I/R treatment, yet its trend could be improved by sulodexide treatment. The glutathione level reflects the body's ability to clear out oxygen free radicals in the body, thereby protecting the structure and functional integrity of cell membranes; MDA can increase the level of peroxides in the body, resulting in cell damage, and MPO is unique to neutrophils, indicating that cell-mediated inflammatory responses are suppressed.

In the present study, inhibited BAX and BAD activity and escalated BCL-2 and BCL-xL expression levels in in vivo rat models collectively verified sulodexide being the mitigator of cellular late apoptosis, which was found to be extensively involved in limb I/R injury conditions in our previous studies. Besides, sulodexide also restrained caspase-3 activation and the expression of BAX and BAD in HK2 cells under H/R-induced injury. In conclusion, sulodexide might inhibit kidney cell via directly modulating the expression of apoptosis-related proteins.

There are also some limitations to this study. A multitude of likely signaling transduction pathways supposedly run through the whole damage-repair process, yet most of which have not been clearly clarified so far. For example, the mitogen-activated protein kinase/extracellular signal-regulated kinase (MAPK/ERK) signaling pathway serves as a crucial pathway in I/R-induced cellular apoptosis [31]. In future research, the research avenue into the activity MAPK/ERK signaling pathway under sulodexide-dominated I/R therapy would be what we are eager to shed light on.

In conclusion, sulodexide can alleviate renal injury caused by hind-limb I/R injury by enhancing cell proliferation and exhibiting anti-inflammatory, antioxidative stress, antiapoptosis, and anticoagulation effects. This indicates that sulodexide may be a potential agent for renal detriment following hind-limb I/R injury prevention and treatment. Even though all above establishes solid foundation for sulodexide to be a promising agent on prophylactic and therapeutic applications towards renal injury and systemic aggravation due to hind-limb I/R injury, whether sulodexide administration can effectively reach ideal clinical outcomes and how to make it optimally realize its unique efficacy remain to be determined and deserve endless attention and endeavor in the future.

## Data Availability

The data used to support the findings of this study are available from the corresponding author upon request.
